# The effect of pupil transmittance on axial resolution of reflection phase microscopy

**DOI:** 10.1038/s41598-021-02188-0

**Published:** 2021-11-23

**Authors:** Min Gyu Hyeon, Kwanjun Park, Taeseok Daniel Yang, Taedong Kong, Beop-Min Kim, Youngwoon Choi

**Affiliations:** 1 Interdisciplinary Program in Precision Public Health, Korea University, Seoul, 02841 Korea; 2 Department of Bioengineering, Korea University, Seoul, 02841 Korea; 3 School of Biomedical Engineering, Brown University, Providence, RI 02912 USA

**Keywords:** Optics and photonics, Microscopy, Interference microscopy

## Abstract

A reflection phase microscope (RPM) can be equipped with the capability of depth selection by employing a gating mechanism. However, it is difficult to achieve an axial resolution close to the diffraction limit in real implementation. Here, we systematically investigated the uneven interference contrast produced by pupil transmittance of the objective lens and found that it was the main cause of the practical limit that prevents the axial resolution from reaching its diffraction limit. Then we modulated the power of illumination light to obtain a uniform interference contrast over the entire pupil. Consequently, we could achieve an axial resolution fairly close to the diffraction limit set by the experimental conditions.

## Introduction

Phase measurement based on interferometric detection has been widely exploited to investigate the physical properties of translucent specimens. In the past decades, a variety of approaches have been introduced for precision measurements of biological samples such as cell dry mass^1–4^, biomechanics associated with cell status^5–11^, and scattering properties of tissues^12–15^ in either transmission or reflection geometry. Of the two configurations, the reflection phase microscopy has been more suitable for topographic measurements because it is free from the ambiguity involved in the interpretation of the transmission phase. For instance, the fine motion of the outmost membrane of a cell can be identified without the uncertainty caused by unidentified refractive indices of multiple intracellular structures^16–20^.

To specify a target depth among multiple layers, it is essential for the reflection measurement to be equipped with an appropriate axial resolution. One way of achieving the depth selectivity is by using temporal gating generated by broad spectra of light sources. Usually, pulsed lasers^18^ or thermal light sources^19,21–23^ are employed to obtain the axial resolution corresponding to their coherence lengths. The second stream is utilizing spatial coherence of light where the llumination light with short spatial coherence in 3-dimensional (3-D) space is generated by destroying the spatial mode of a laser beam^17,24^. In this case, the depth selectivity is determined by the spatial decorrelation length of the speckle field rather than the intrinsic coherence length of the light source. These methods have shown the depth sectioning and demonstrated 3-D imaging of an object, however, they have certain limitations such as either the lack of axial resolution or an insufficient imaging speed.

To overcome the limitations in the reflection measurement, an RPM employing spatio-temporal coherence of light was demonstrated^25^. By combining the two gating mechanisms together with a high-power laser, both requirements sufficient for observing membrane fluctuation of living eukaryotic cells were satisfied. In this demonstration, however, being equipped with a high power light source was essential due to the huge loss of light power caused by the rotating diffuser generating a time-varying speckle field. To resolve this problem, a novel gating mechanism called the successive accumulation of interferograms (SAI) was proposed^26^. In this development, multiple interferograms were cumulatively acquired in a single exposure of a camera to form a depth selectivity while each being generated by various angular plane waves. Since the SAI process worked with a plane wave illumination, the loss of light power was minimized, and thus it demonstrated fast image acquisition even with 40 times less light power than the former demonstration.

The RPMs including the SAI, which rely on the spatial coherence, have demonstrated good imaging performances and shown an axial resolution near the one expected by theory, but it has been very difficult to reach the limit, particularly for high numerical aperture (NA) imaging, in real implementations. The main reason for the discrepancy is the angle-dependent transmittance of an objective lens that causes an unequal interference contrast in *k*-space. This results in a decrease in the achieved axial resolution.

In this study, we investigated the transmittance of objective lenses in an RPM employing Linnik-type interferometry. To explore its effect on the interference between the two beams in the interferometry over the NA of the objective lens, the illumination port of the RPM was constructed with the SAI configuration. Thus the objective’s pupil was addressed point-by-point by steering a plane wave. Then we found that the decrease in the interference contrast at a higher NA side was the main cause of the degradation in the axial resolution. For flattening the interference over the entire NA of the objective lens, the power of the input plane wave was modulated so that the degradation of the interference at a higher NA side was compensated for. The elaborate manipulation of the pupil function of the objective lens resulted in uniform interference over the entire NA of the objective lens, and thus the axial resolution of the RPM was improved beyond the practical limit set by the variation of angular transmittance of the objective lenses. We experimentally achieved an axial resolution of about 758 nm, which was very close to the theoretical value based on our experimental conditions, with an error rate of 1.06%.

## Results

### Experimental setup

**Figure 1:**
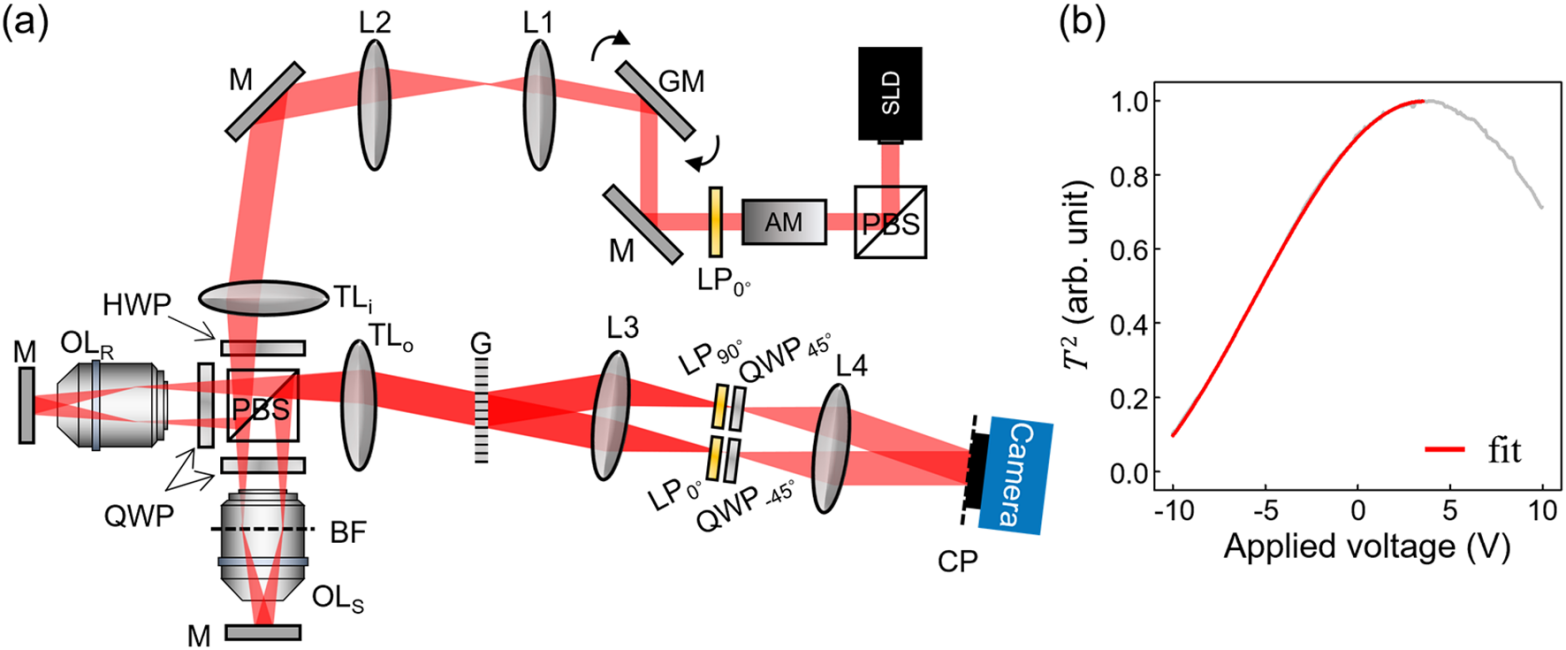
Experimental setup and amplitude modulation. (**a**) Schematic diagram of the experimental setup. AM: amplitude modulator; M: mirror; L1–L4: lenses; GM: galvano mirror; ${\text{LP}}_{{0^{\circ}}}$: output polarizer; ${\text{TL}}_{{\text{i}}}$ and ${\text{TL}}_{{\text{o}}}$: tube lenses for input and output ports; HWP: half-wave plate; PBS: polarizing beam splitter; QWP: quarter-wave plate; ${\text{OL}}_{{\text{R}}}$ and ${\text{OL}}_{{\text{S}}}$: objective lenses for reference and sample arms; BF: back focal plane of objectives; G: diffraction grating; ${\text{LP}}_{\alpha }$: linear polarizer with $\alpha$-deg rotation angle. (**b**) Output intensity response of the AM to the driving voltage.

The experimental schematic is depicted in Fig. 1a. The microscope was based on Linnik-type interferometry employing an off-axis configuration in the detection port. The basic microscope structure is similar to the one reported in our previous demonstration using the SAI process^26^. The main difference between the present study and the previous study was that an electro-optic amplitude modulator (AM, Thorlabs, EO-AM-NR-C1) and a linear polarizer (${\text{LP}}_{0^{\circ}}$) with the polarization axis aligned vertically were used in the present study. The vertically-polarized (v-pol.) light from a superluminescent diode (SLD, Superlum) with a center wavelength $\lambda_{0}$ = 795 nm and a spectral bandwidth $\Delta \lambda$ = 15 nm was reflected off a polarizing beam splitter (PBS) to purify the polarization. The output intensity through ${\text{LP}}_{0^{\circ}}$ was adjusted by applying a voltage to the AM. The v-pol. wave transmitted through the AM was incident on the sample and the reference arms via another PBS and a half-wave plate (HWP) for power rebalancing between both arms. A 2-D galvanometer mirror (GM, Saturn 1B, Pangolin Laser System) was placed in the beam path to steer the illumination angle on both the sample and the reference planes. The GM and the AM were synchronized such that the light intensity was modulated depending on the illumination angle.

The intensity of the input wave to the system was controlled by the AM in the beam path. Since the polarization axis of the light was linearly rotated by the AM according to the applied voltage, the output intensity through the ${\text{LP}}_{0^{\circ}}$ had a typical voltage response as $T^{2} (V) = \sin^{2} (\gamma_{0} - \kappa V)$, where $T$ is the amplitude modulation transmittance, $\gamma_{0}$ and $\kappa$ were characteristic constants of the AM. The measured intensity depending on the applied voltage is shown in Fig. 1b. The red line in Fig. 1b represents a fitting curve in a range of − 10 to 3.52 V, which corresponds to the minimum-to-maximum intensity range. It also corresponds to the voltage range available for the following experiments.

Here we briefly introduce the working principle, SAI of our microscope. Due to the identical optical configuration in both arms, the two beams are synchronously steered in their angles on the sample and the reference planes. Within a single exposure of a camera, we scan the beam such that the focused spot sweeps the entire back focal plane (BF) with a uniform sampling density, which results in full coverage of the NA of the objective lenses. After being reflected off at the sample and the reference planes, the two beams co-propagate through the detection port and are slit into multiple diffraction orders at the grating (G). Among the many orders, only the 0-th and the 1-st orders are chosen by a beam block (not shown in Fig. 1a). Due to the cross configuration of the linear polarizers (${\text{LP}}_{0^{\circ}}$ and ${\text{LP}}_{{90^{\circ}}}$) at the Fourier plane, only one beam (the sample or the reference beam) can pass through the polarizer in each order. Subsequently, the polarization of the two beams is matched by the two quarter-wave plates (${\text{QWP}}_{{45^{\circ } }}$ and ${\text{QWP}}_{ - 45^{\circ} }$). The two beams meet at the camera at a fixed angle defined by the grating and then form an interferogram. Since we keep the camera open during the angle scanning, multiple interferograms are accumulated. Interestingly, we found that the integration of multiple interferograms results in a coherent addition of electric fields of the sample beam. While accumulating the interferograms, the angle-dependent phase shift generated by the optical path difference between the two arms significantly reduces the fringe contrast and quickly washes out the defocused information. This is the origin of the depth selectivity produced by the SAI process and its ability to reject the out-of-focus information, i.e., the axial resolution of the system, thoroughly depends on the uniformity of the interference contrast over all the spatial frequency components.

### Amplitude modulation coefficient

We consider a simple model for the problem of angle-dependent non-uniform transmittance. To account for the reflection configuration, where the beam passes the same objective lens twice, it is necessary to consider the double transmission as a one way traveling. Assume that $t_{S} (\vec{k})$ and $t_{R} (\vec{k})$ are the angle-dependent amplitude transmittances in the double transmission situation for the sample and the reference beams, respectively (See more details about the amplitude transmittance in Supplementary Note 1). The contribution of the input wave with $\vec{k}$ to the interferogram at the camera then can be expressed as

1 $$I(\vec{k};\Delta z) = \left| {t_{S} (\vec{k})E_{S}^{0} + t_{R} (\vec{k})E_{R}^{0} e^{{i(2k_{z} \Delta z + k_{x}^{R} x + \phi_{u} (\vec{k}))}} } \right|^{2} ,$$

for the case of arbitrary pathlength difference $\Delta z$ between the two arms, where $E_{S}^{0}$ and $E_{R}^{0}$ are constant input amplitudes of the sample and the reference beam, respectively, $k_{z}$ is the *z*-component of the wavevector $\vec{k}$, $k_{x}^{R}$ is the wavevector caused by the off-axis configuration along the *x*-axis, and $\phi_{u} (\vec{k})$ is the angle-dependent phase mismatch between the two waves. As a special case, when $\Delta z = 0$, the interferogram is further simplified as

2 $$I(\vec{k}) = I_{S} + I_{R} + 2\eta (\vec{k})\left| {E_{S}^{0} } \right|\left| {E_{R}^{0} } \right|\cos \left( {k_{x}^{R} x + \phi_{u} (\vec{k})} \right),$$

where $I_{S,R} = t_{S,R}^{2} \left| {E_{S,R}^{0} } \right|^{2} = t_{S,R}^{2} I_{S,R}^{0}$ with $I_{S,R}^{0}$ being the input intensity, and $\eta (\vec{k})$ is the interference contrast given by $\eta (\vec{k}) = t_{S} t_{R}$. In a standard phase retrieval algorithm, the corresponding complex field image is obtained by the Hilbert transform of the interferogram in Eq. (2). At a specific illumination with $\overrightarrow {k}$, the magnitude of the interference is determined by the term of $2\eta (\vec{k})\left| {E_{S}^{0} } \right|\left| {E_{R}^{0} } \right|$ in Eq. (2). Since $\eta (\vec{k})$ depends on $t_{S} (\vec{k})$ and $t_{R} (\vec{k})$ in general, angle-dependent transmittance is the main factor determining the angle-dependent interference contrast in the SAI configuration.

To compensate for the varying $\eta (\vec{k})$ and make the interference contrast uniform in the k-space, the input amplitude was modulated. Consider that the input wave was controlled by the AM with the amplitude modulation transmittance *T* so that the output amplitude was modulated as $E_{S,R}^{M} = TE_{S,R}^{0}$, then the interferogram in Eq. (2) was also modulated as $I^{M} (\vec{k}) = T^{2} I(\vec{k})$ with $I^{M}$ being the interferogram after the modulation. Accordingly, the term for the interference contrast was also modulated as $2T^{2} \eta (\vec{k})\left| {E_{S}^{0} } \right|\left| {E_{R}^{0} } \right|$. To obtain a uniform contrast independent of illumination wavevector $\vec{k}$, $T^{2}$ needs to vary so that it always satisfies the condition of $T^{2} \eta (\vec{k}) = \alpha$, where $\alpha$ is a constant determined by considering the operation range of the AM. Thus, the modulation coefficient is given by

3 $$T^{2} (\vec{k}) = \frac{\alpha }{{\eta^{ - 1} (\vec{k})}} = \frac{\alpha }{{t_{S} (\vec{k})t_{R} (\vec{k})}}$$

to compensate for the variation of the angle-dependent interference contrast. Since $t_{S} (\vec{k})$ and $t_{R} (\vec{k})$ are measurable quantities, $T^{2} (\vec{k})$ can be obtained by Eq. (3). Once it is done, the voltage, $V_{c} (\vec{k})$, required for the AM control can be determined by comparing $T^{2} (\vec{k})$ with the voltage response of the AM $T^{2} (V)$ in Fig. 1b.

### Measurement of non-uniform transmittance

**Figure 2:**
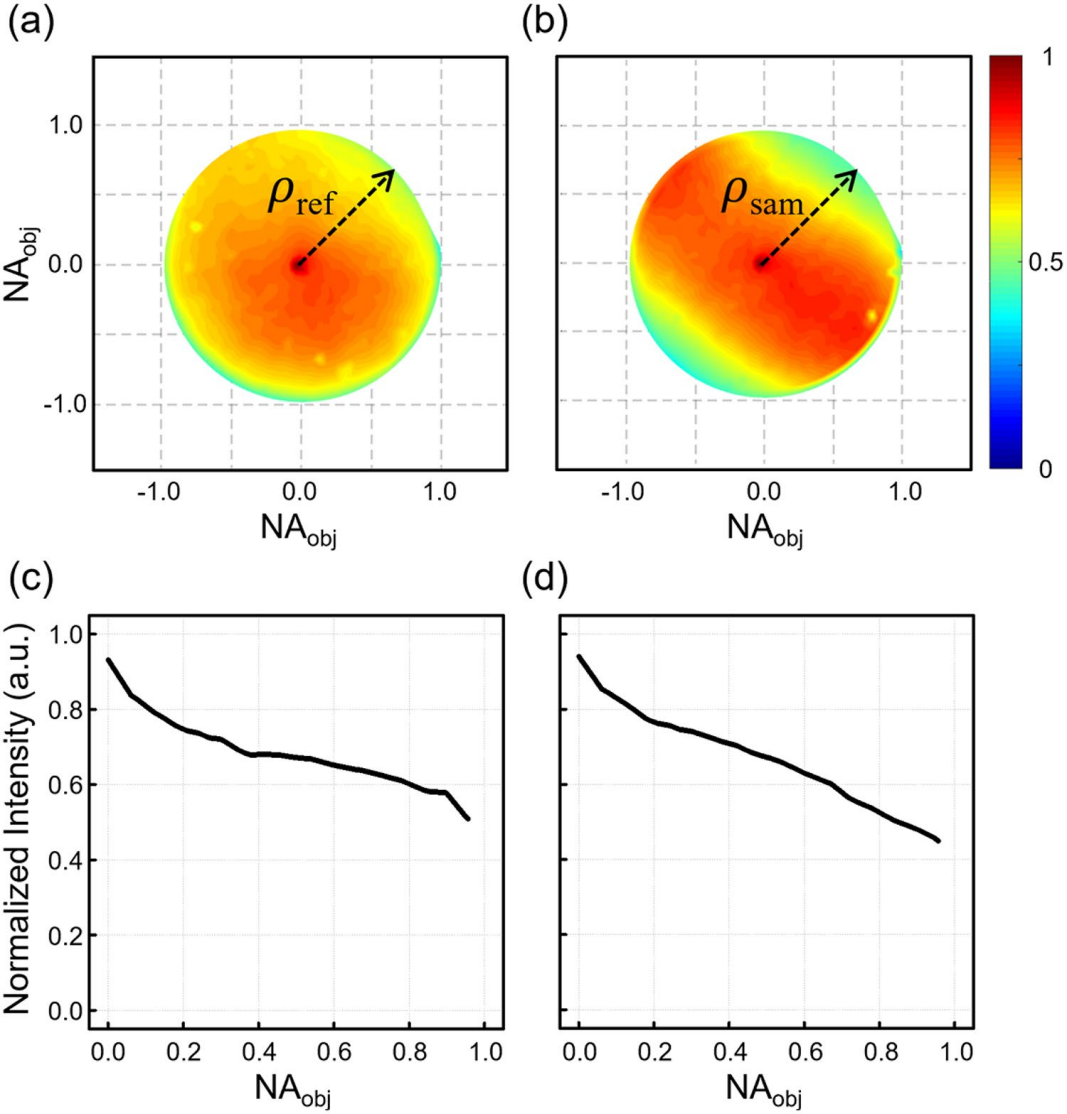
Intensity transmission through objective lenses. (**a**) $t_{R}^{2}$ for the reference objective. (**b**) $t_{S}^{2}$ for the sample objective. (**c**, **d**) Line profiles along dashed lines in (**a**) and (**b**), respectively.

To investigate the tendency of light transmission through the two objective lenses depending on the incident angle, we used 1000 plane waves uniformly covering the back focal plane of the objective lens. Each intensity image was captured by the camera. Its average value over the entire area was obtained as the transmittance for the illumination angle. All transmittance values were mapped on the back focal plane of the objective lens as shown in Fig. 2a,b for the reference and the sample beam, respectively. Line profiles along the radial direction $\rho_{ref}$ and $\rho_{sam}$ represented by black arrows in Fig. 2a,b are presented in Fig. 2c,d. The transmittance was gradually attenuated from low to high spatial frequency. The power of light was reduced to about 53.7% in the reference arm and about 47.8% in the sample arm at the edges of the back focal planes. Detailed analysis and extra experiments for the transmittance measurement are presented in Supplementary Note 1 and Supplementary Note 2.

**Figure 3:**
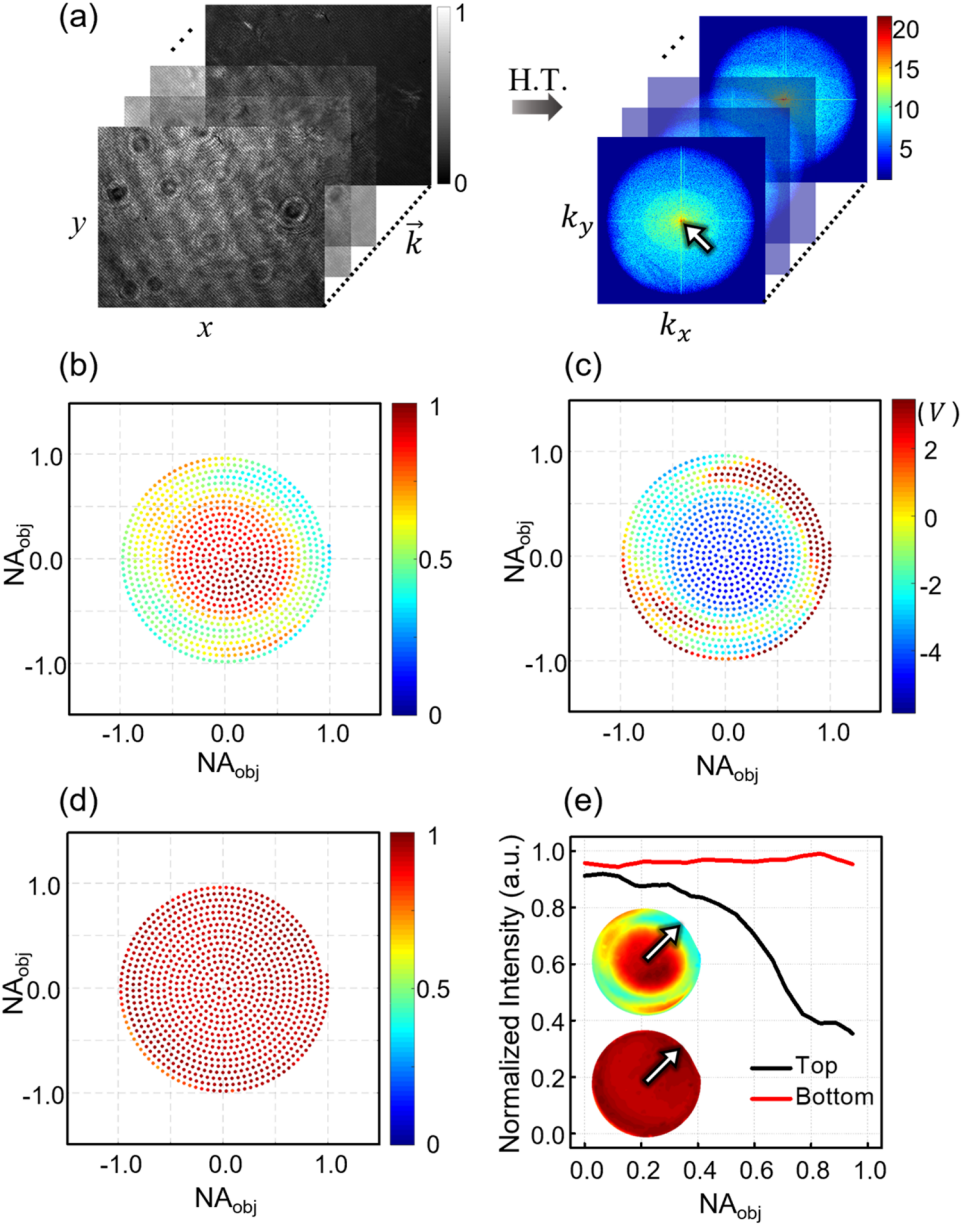
Uniformity of the interference contrast. (**a**) Raw interferograms at various illumination angles and corresponding angular spectra in *k*-space. The center peak in the k-space is indicated by a white arrow. (**b**) Normalized $\eta (\vec{k})$ mapped in the NA of the objective lens without the AM control. (**c**) Driving voltage required for the AM control. (**d**) Normalized $\eta (\vec{k})$ with the AM control. (**e**) Section profiles along the direction along the white arrows in insets. Insets: interpolated images of (**b**) top and (**d**) bottom.

Since these two distributions were neither flat nor identical to each other, the interference strength formed by both waves was affected. Thus, its uniformity over the illumination NA was ruined. To investigate the actual interference contrast, interferograms were obtained at 1000 different illumination angles over the whole NA of the objective lens. Representative interferograms and their angular spectra are shown in Fig. 3a. For each spectrum, the peak value at the center denoted by the white arrow was obtained. This corresponded to an empty interference strength between the two waves. Note that although the illumination angle varied according to the scan, the DC peak in the spectra was static at the center because the relative angle between the two illuminations was fixed by synchronous scanning on both arms.

During this measurement, the AM was driven by a fixed voltage, $$V_{i}$$ =  − 2.5 V, which was chosen within the entire driving range such that the AM could either amplify or attenuate the output power to flatten the interference contrast. Figure 3b shows an uncontrolled interference contrast, i.e., $$\eta (\vec{k})$$, observed over the NA of the objective lens, which was normalized to the highest value. It showed an abnormal behavior, fluctuating between 1 and 0.2. This non-uniform distribution caused degradation of the axial resolution in the SAI process. To restore the uniformity of the interference contrast, we first obtained the driving voltage required for the AM control. By Eq. (3) together with the measured $$\eta (\vec{k})$$ in Fig. 3b, $$T^{2} (\vec{k})$$ was determined and then converted to $$V_{c} (\vec{k})$$$$,$$ the driving voltage for compensating the fluctuation of the interference contrast, using the $$T^{2} (V)$$ curve in Fig. 1b. The obtained $$V_{c} (\vec{k})$$ is presented in Fig. 3c. Note that the AM driving voltage is set to amplify (attenuate) the intensity for the high (low) angle illumination with a low (high) interference contrast.

Next, $V_{c} (\vec{k})$ was applied to the AM synchronously with GM scanning. After compensation by AM control, the inference contrast became almost uniform over the entire NA as shown in Fig. 3d, where the map was normalized with the highest value. Insets in Fig. 3e show interference contrast maps in Fig. 3b,d with interpolations for a smooth visualization. Section profiles along arrows in the insets show variations of the flatness of the interference contrast before and after AM control. As seen in these profiles, after the compensation, the interference contrast could keep the initial value almost up to the edge of the highest NA.

### Enhancement of axial resolution by amplitude modulation

To demonstrate the enhancement of the axial resolution after the AM control, the SAI process was investigated. In our previous work, typically *N* = 200 different plane waves over the entire NA of the objective were used to generate the SAI process^26^. Here in this study, we used *N* = 1000 just for the presentation purpose of the smooth maps in Figs. 2 and 3. With this change, the imaging speed reduces from 100 to 20 fps due to the limit of the galvanometer mirrors. According to our previous study, the axial resolution formed by the SAI is not highly sensitive to the number of plane waves *N* used in a single exposure, rather it depends on the NA extended by the angular scanning. The quality of optical sectioning also does not change much with *N* more than 200. Thus, we can use *N* = 200 whenever we need and recover the original acquisition speed of 100 fps without compromising the imaging resolution.

Before the main discussion, we will briefly explain the generation of optical sectioning by the SAI process. With the accumulation of angle-dependent interferograms during a single exposure, the final interferogram at the camera is obtained as,

4 $$I_{SAI} \left( {\Delta z} \right) = \sum\limits_{i}^{N} {I\left( {\vec{k}_{i} ;\Delta z} \right)}$$

where $I(\vec{k}_{i} ;\Delta z)$ is the *i*-th angle-dependent interferogram shown in Eq. (1). These individual interferograms generated with $\Delta z = 0$ have an identical pattern. Thus, the contrast of $I_{SAI} (\vec{k}_{i} ;\Delta z = 0)$ is maintained even when all interferograms are accumulated in a single exposure. However, with $\Delta z \ne 0$, each interference pattern changed due to an additional angle-dependent phase shift by $\exp (i2k_{z} \Delta z)$ in Eq. (1). As a consequence, the final interferogram $I_{SAI} (\vec{k}_{i} ;\Delta z \ne 0)$ lost the contrast and the signal was attenuated accordingly. This was the origin of the generation of axial resolution in the SAI. More details about the SAI process can be found in a previous study^26^.

**Figure 4:**
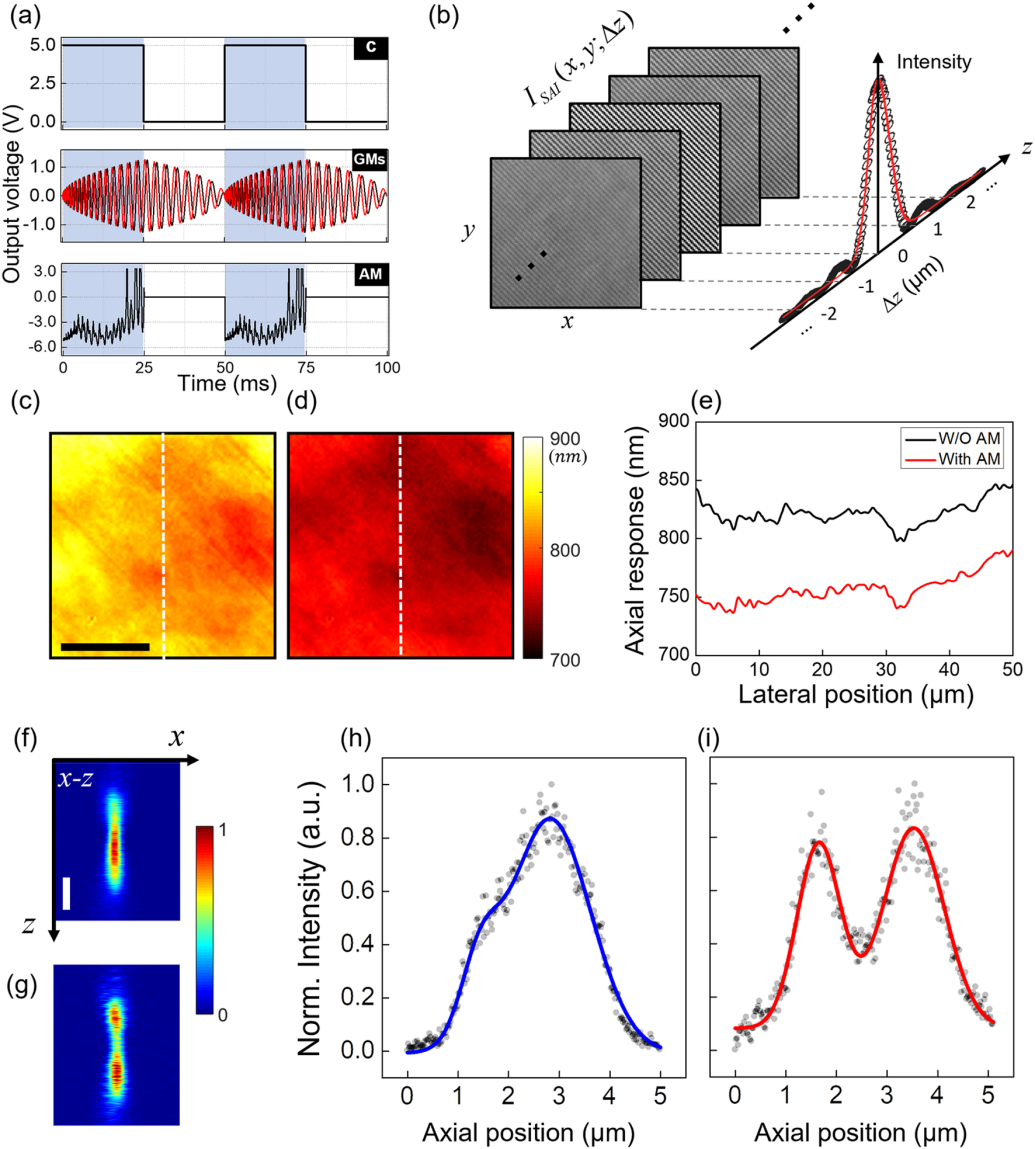
Enhancement of the axial resolution. (**a**) Time sequences of external triggers for the operation of devices in the setup. C: camera; GMs: Galvanometer mirrors; AM: amplitude modulator. (**b**) Measured interferograms (left) and intensity of the image (right) as a function of $\Delta z$. The red line is a Gaussian fit. (**c**) Map of the measured axial resolution over the FOV before the AM control. (**d**) The same as (**c**) after the AM control. Scale bar: 20 μm. (**e**) Section profiles along the dashed lines in (**c**) and (**d**). (**f**, **g**) 2-D depth (*x–z* plane) images of a cluster of two magnetic beads suspended in an agarose gel without (**f**) and with (**g**) the AM control. Scale bar: 1 μm, colorbar: normalized intensity. (**h**) Projected profile of (**f**) onto the *z*-axis. (**i**) The same as (**h**) of (**g**). The solid lines in (**h**) and (**i**) are fitting curves.

We investigated the effect of AM control on the axial resolution in the SAI process. Figure 4a shows time sequence of the camera (C), GMs, and AM for the measurement. The compensation voltage in Fig. 3c was applied to the AM synchronously with GM steering. While controlling GMs and the AM with 1000 steps, the camera opened and kept a single exposure, which was driven by an external TTL signal with a duty cycle of 50% and a speed of 50 ms, accumulating 1000 angle-dependent interferograms.

To measure the axial resolution, $I_{SAI} (\Delta z)$ was measured at different $\Delta z$ using a flat mirror as a sample. The sample mirror was placed on a computer-controlled motorized translation stage (N-565, Physik Instrumente). $\Delta z$ was varied by moving the stage by a total distance of 6 μm with an interval of 30 nm. Thus, $I_{SAI} (\Delta z)$ at 200 different $\Delta z$ was recorded. Among all acquired $I_{SAI} (\Delta z)$, representative interferograms near $\Delta z = 0$ are shown in Fig. 4b. After the image processing, the intensity value at a certain point in the image of the sample mirror as a function of $\Delta z$ is shown as black circles in Fig. 4b. The red line represents Gaussian fitting. Due to the SAI process, the interference image had the highest intensity at $\Delta z = 0$, whereas it decreased as $\Delta z$ moved away from the origin. The full width at half maximum (FWHM) of the Gaussian fit for the intensity profile was approximately 826 nm without AM control. We mapped FWHMs measured at every point in the imaging area of 50 × 50 μm^2^ as shown in Fig. 4c. The mean value of the axial resolution over the entire field of view (FOV) was measured to be 825.7 ± 18.0 nm. Considering that the theoretical expectation of the axial resolution was 750 nm in our experimental conditions, it was broadened by about 10% due to non-uniform and non-identical transmissions through the two objective lenses.

Next, we controlled the AM according to the compensation voltage shown in Fig. 3c so that the interference contrast became uniform over the entire *k*-space. After the AM control, we measured the axial resolution over the entire FOV as presented in Fig. 4d. The mean value of the axial resolution was measured as 758 ± 13.4 nm, which was fairly close to the expectation value (750 nm) mentioned above. The difference is highlighted in Fig. 4e, where the section profiles along the vertical dashed lines in Fig. 4c,d are presented in the black and the red lines, respectively. We also numerically investigated the relation between the uneven transmittance of the objective lens and the axial resolution in Supplementary Note 3.

In order to verify the effect of the AM control in a more realistic situation, we made a phantom using an agarose gel as a host medium and magnetic beads as scatterers. The size of the beads ranged from 400 to 690 nm. We sought a cluster of two beads that were close to each other along the *z*-axis such that the individual particles were not clearly resolved. Then we acquired multiple depth images for the cluster and created 2-D (*x–z*) images without and with the AM control as shown in Fig. 4f,g, respectively. Before the AM control, the identification of the two beads was very unclear in Fig. 4f, but after the AM control, the existence of the two particles was well verified in Fig. 4g. For further analysis, we projected the 2-D images in Fig. 4f,g onto the *z*-axis and produced the plots for their 1-D axial profiles as presented in Fig. 4h,i, respectively. The solid lines are fitting curves with two-peak Gaussian functions. As seen in the plots, the AM control made our microscope clearly distinguish the individual beads otherwise, those could not be resolved. When comparing the two cases, it was verified that our method indeed can enhance the resolving power of the RPM along the axial direction. For more details about the phantom experiment, see Supplementary Note 4.

Even considering the size of the beads, the widths of the distributions were axially elongated to a certain degree compared to the axial resolution measured in the ideal situation as shown in Fig. 4c,d. This was because of the aberrated effect induced by the agarose gel of the test phantom. In the sample, the beads were embedded in the agarose gel while there was only water in the reference plane, which produced a slight refractive index difference between the two arms causing a broadening of the object profiles along the axial direction. The effect of the sample-induced aberration will be minimal for a sample that can be immersed in the same medium as that in the reference arm, such as single cells. But this can be increased when imaging a volumetric sample with a refractive index different from water, such as bulk tissues.

## Discussion and conclusion

We investigated the pupil transmittance of the objective lens and found that it reduced at high NA region. The uneven trasnsmittance resulted in the unequal interference contrast of the two beams and thus became a main cause of degradation of the axial resolution in an RPM. To resolve the problem, the interference contrast was flattened by the AM control of the laser power of illumination beam. As a result of the AM control, the interference contrast of the two beams became uniform over the entire pupil of the objective lense. Consequently, the axial resolution quite close to the theoretical limit could be observed. Here in this study, we investigated the pupil transmittance and also observed the enhancement of the axial resolution by the AM control with an RPM working with the SAI precess. This was simply because that the SAI microscope enabled us to precisely address the pupil plane without major modification. Since the SAI configuration was already equipped with a 2-D galvanometer mirror for scanning the illumination beam over the entire pupil plane. It allowed to easily examine the response of the objective lens to the well-defined spatial frequency and thus to identify the main cause of the degradation of the axial resolution. Thus, in the present study, the SAI configuration was utilized as a precise tool for the investigation of the system response.

Although the SAI configuration was essential for the assessment of the system’s response, but it was inessential for the correction of the pupil function. Since most RPMs utilizing large pupils for superior optical sectioning have the similar degradation of the axial resolution from the idea limit, the use of our method is not simply limited to the SAI microscope. Rather its utility can be easily extended to RPMs working with the spatial coherence of light just by employing a compensation plate at the objective’s pupil plane, which can flatten the angle-dependent transmittance. Therefore, our method can be utilized to improve the performance of RPMs beyond the limit set by the practical environments.

## Supplementary Information

Supplementary Information.

## References

[1] Popescu G (2008). Optical imaging of cell mass and growth dynamics. Am. J. Physiol. Cell Physiol..

[2] Mir M (2011). Optical measurement of cycle-dependent cell growth. Proc. Natl. Acad. Sci. U. S. A..

[3] Sung YJ (2013). Size homeostasis in adherent cells studied by synthetic phase microscopy. Proc. Natl. Acad. Sci. U. S. A..

[4] Sung Y, Choi W, Lue N, Dasari RR, Yaqoob Z (2012). Stain-free quantification of chromosomes in live cells using regularized tomographic phase microscopy. PLoS ONE.

[5] Popescu G (2008). Imaging red blood cell dynamics by quantitative phase microscopy. Blood Cell Mol. Dis..

[6] Park Y (2010). Measurement of red blood cell mechanics during morphological changes. Proc. Natl. Acad. Sci. U. S. A..

[7] Hosseini P (2016). Cellular normoxic biophysical markers of hydroxyurea treatment in sickle cell disease. Proc. Natl. Acad. Sci. U. S. A..

[8] Popescu G, Ikeda T, Dasari RR, Feld MS (2006). Diffraction phase microscopy for quantifying cell structure and dynamics. Opt. Lett..

[9] Park YK (2008). Refractive index maps and membrane dynamics of human red blood cells parasitized by *Plasmodium falciparum*. Proc. Natl. Acad. Sci. U. S. A..

[10] Popescu G (2006). Optical measurement of cell membrane tension. Phys. Rev. Lett..

[11] Li Y, Fanous MJ, Kilian KA, Popescu G (2019). Quantitative phase imaging reveals matrix stiffness-dependent growth and migration of cancer cells. Sci. Rep..

[12] Ding H (2011). Measuring the scattering parameters of tissues from quantitative phase imaging of thin slices. Opt. Lett..

[13] Lee M (2016). Label-free optical quantification of structural alterations in Alzheimer’s disease. Sci. Rep..

[14] Wang Z, Popescu G, Tangella KV, Balla A (2011). Tissue refractive index as marker of disease. J. Biomed. Opt..

[15] Ding H, Wang Z, Nguyen F, Boppart SA, Popescu G (2008). Fourier transform light scattering of inhomogeneous and dynamic structures. Phys. Rev. Lett..

[16] Choi Y (2014). Dynamic speckle illumination wide-field reflection phase microscopy. Opt. Lett..

[17] Redding B, Bromberg Y, Choma MA, Cao H (2014). Full-field interferometric confocal microscopy using a VCSEL array. Opt. Lett..

[18] Yaqoob Z (2011). Single-shot full-field reflection phase microscopy. Opt. Express.

[19] Yamauchi T, Iwai H, Yamashita Y (2011). Label-free imaging of intracellular motility by low-coherent quantitative phase microscopy. Opt. Express.

[20] Singh VR (2019). Studying nucleic envelope and plasma membrane mechanics of eukaryotic cells using confocal reflectance interferometric microscopy. Nat. Commun..

[21] Li XH (2006). Full-field quantitative phase imaging by white-light interferometry with active phase stabilization and its application to biological samples. Opt. Lett..

[22] Iwai H (2004). Quantitative phase imaging using actively stabilized phase-shifting low-coherence interferometry. Opt. Lett..

[23] Yamauchi T, Iwai H, Miwa M, Yamashita Y (2008). Low-coherent quantitative phase microscope for nanometer-scale measurement of living cells morphology. Opt. Express.

[24] Somekh MG, See CW, Goh J (2000). Wide field amplitude and phase confocal microscope with speckle illumination. Opt. Commun..

[25] Choi Y (2018). Reflection phase microscopy using spatio-temporal coherence of light. Optica.

[26] Hyeon MG (2019). Reflection phase microscopy by successive accumulation of interferograms. ACS Photonics.

