# Supplementary material for: The effect of pupil transmittance on axial resolution of reflection phase microscopy
Source: Sci Rep. 2021 Nov 23;11:22774. doi: 10.1038/s41598-021-02188-0 (PMC8610988; doi:10.1038/s41598-021-02188-0)
Supplement: Supplementary file 1 — Supplementary Information. [file 41598_2021_2188_MOESM1_ESM.pdf]

The effect of pupil transmittance on axial resolution of reflection phase microscopy

Min Gyu Hyeon[1], Kwanjun Park[1], Taeseok Daniel Yang[1,2], Taedong Kong[1], Beop-Min Kim[1,3,4], and Youngwoon Choi[1,3,5]

[1]Department of Bioengineering, Korea University, Seoul 02841, Korea
[2]School of Biomedical Engineering, Brown University, Providence, Rhode Island 02912, USA
[3]Interdisciplinary Program in Precision Public Health, Korea University, Seoul 02841, Korea
Corresponding authors: [4]bmk515@korea.ac.kr and [5]youngwoon@korea.ac.kr

Supplementary Note 1

Transfer function of an objective lens

As an output response of the system, we show the intensity transmittances of the objective lenses in Fig. 2 in the main text. Here we present more details about the measurements. The experimental schematic is drawn in Fig. S1 (a). As described in the main text, we illuminate a plane wave with specific wavevector (k_x, k_y) through the objective lens. The plan wave passes the back focal (BF) plane of the objective lens through a single point as shown in Fig. S1 (a). Then the wave becomes an angular plane wave at the mirror located at the focal plane of the objective lens. Subsequently, the wave is reflected off at the mirror surface following the law of reflection thus returns back through the BF again via another single point $(-k_x, -k_y)$. Consequently, the amplitude of the returning wave via the double-pass geometry of the setup and then can be expressed as

$$t = H(k_x, k_y)H(-k_x, -k_y), \tag{S1}$$

where $H(k_x, k_y)$ is the single-pass amplitude transfer function (ATF) of the objective lens, which is also known as a coherent transfer function (CTF) [1]. As the response of the system, we measured the intensity at the camera. Thus the quantity which is presented in Fig. 2 is

$$|t|^2 = |H(k_x,k_y)H(-k_x,-k_y)|^2 . \tag{S2}$$

For simplicity of the expression, we assume that the CTF of the objective lens has an angular symmetry, i.e., $H(k_x,k_y) \approx H(-k_x,-k_y)$. Then we can simplify the expression in Eq. (S2) as

$$|t|^2 \approx |H(k_x,k_y)|^4 . \tag{S3}$$

In the main text, the intensity transmittance of the sample (reference) objective lens is defined as $t_S^2 \equiv |t_S|^2 \approx |H_S(k_x,k_y)|^4$ ($t_R^2 \equiv |t_R|^2 \approx |H_R(k_x,k_y)|^4$). To address the intensity response of the objective lenses over the entire BF, we vary the input wavevector and measure the corresponding output intensities with 1000 different illumination angles. Thus the quantity presented in Fig. 2 in the main text is proportional to quartic order of $|H(k_x,k_y)|$, a common expression of the CTF. For this reason, a small reduction of the amplitude transmittance at the high frequency region by a single-pass transmission can be amplified causing the large decrease in $|H(k_x,k_y)|^4$.

For more direct comparison, we plot the fourth root of the values shown in Fig. 2 (c) and (d), and present the results in Fig. S1 (b) and S1 (d), respectively. Their line profiles along the lines in the figures are also shown in Fig. S1 (d) and S1 (e). Although these results do not show the exact CTFs of the objective lenses, but they provide the averaged single-pass amplitude transfer functions. Comparing the figures in Fig. S1 with those in Fig. 2 in the main text, we can observe that the averaged amplitude transfer functions become flatter and thus look more like a usual CTF.

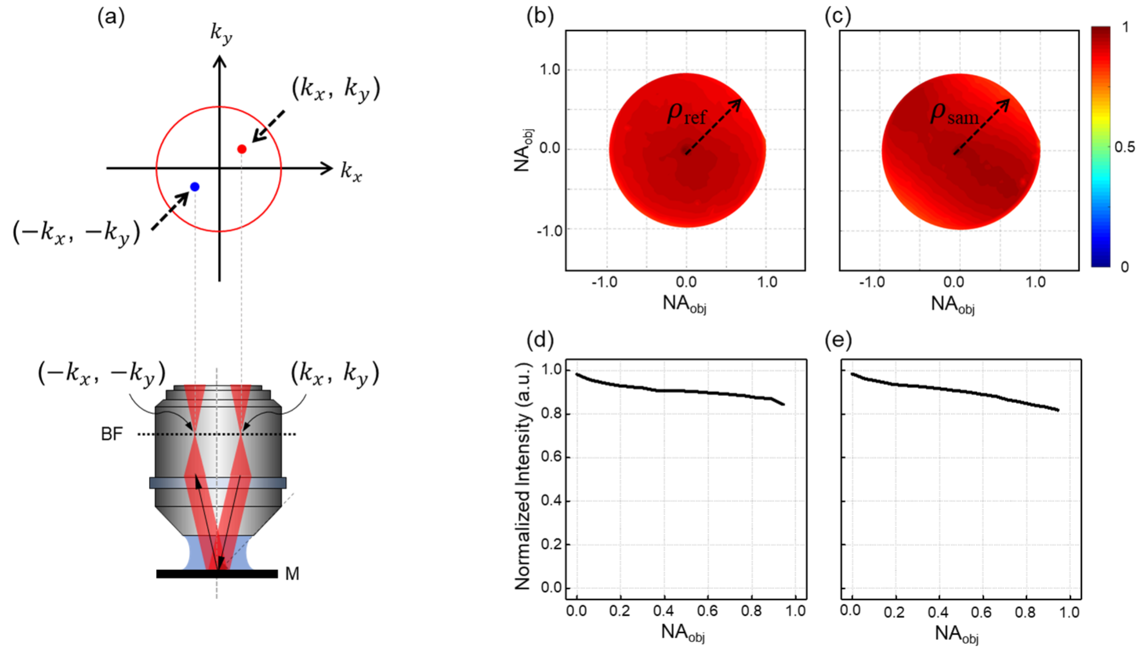

Fig. S1. Double-pass geometry in the measurement of the output response of the system. (a) Schematic of the intensity measurement through the objective lens. BF: back focal plane of the objective lens, M: mirror. For the input wavevector (k_x, k_y), the output wave returns through $(-k_x, -k_y)$ at the BF. (b) Fourth root of the map in Fig. 2 (a) in the main text. This shows the averaged amplitude transfer function of the reference objective lens, $\sqrt{|H_R(k_x,k_y)H_R(-k_x,-k_y)|} \approx |H_R(k_x,k_y)|$. (c) The same as (b), but for the map in Fig. 2 (b) showing $|H_S(k_x,k_y)|$. (d)-(e) Line profiles along the lines in (b) and (c), respectively.

Supplementary Note 2

System response caused by the remaining optics excluding the objective lens

Since we measure the intensity output response of the setup at the camera plane, the measurement shows the overall response of the entire system. In order to examine the effect by the remaining optics excluding the objective lens, we performed an extra experiment.

First, to see the flatness of the input beam, we measured the intensity profiles of the beam with the same scanning configuration at the plane right before the second PBS shown in the setup schematic in Fig. 1 (a) in the main text. The measurement was done either in the sample arm or in the reference arm. The result is shown in Fig. S2 (a) and its profile along the dashed line is presented in the black line in Fig. S2 (d). As seen in the figures, the input beam shows a flat distribution during the angle scanning and there is no significant drop at the higher frequency region.

Next, we removed the objective lens in the sample arm and placed an achromatic lens (f = 30 mm) as a substitution. The achromatic lens was aligned in the similar configuration to the objective

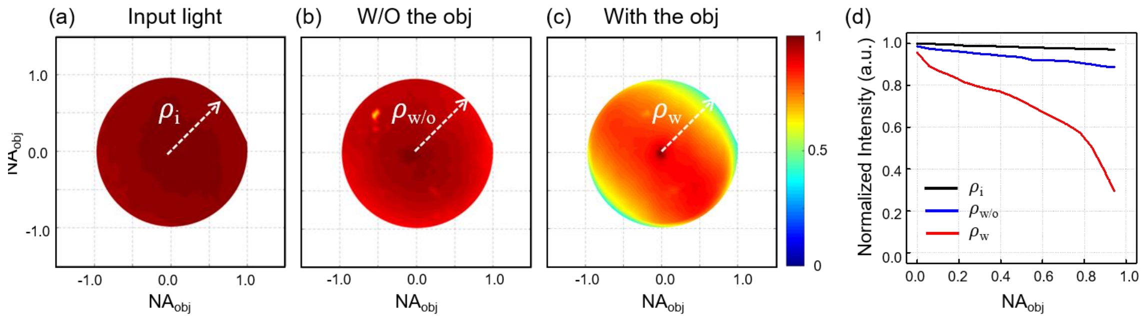

Fig. S2. Intensity response of the system with various configuration. (a) Intensity response of the input beam measured at the plane right before the second PBS in the setup shown in Fig. 1 (a) in the main text. (b) Intensity response of the system measured at the camera plane when placing an achromatic lens instead of the objective lens. (c) Intensity response of the original system, i.e., with the objective lens. In (a)-(c), we measured the intensity during the same scanning configuration and mapped the values in the same NA map of the objective lens for easy comparison. (d) Line profiles along the dashed lines in (a)-

lens so that we could measure the double-pass output intensity while scanning the input beam in the exactly same way. The measured output intensity and its line profile are shown in Fig. S2 (b) and S2 (d) in the blue line, respectively. Although there is a little decrease at the high frequency region, but it is not significant.

Finally, we brought the objective lens back in the setup and measured the output intensity response of the system again. As seen in the experiment in the main text, a significant drop was found as presented in Fig. S2 (c) and S2 (d) in the red line.

From these series of experiments, we confirmed that most contribution of the intensity drop at the higher frequency region came from the objective lens and the effect of the remaining optics, especially the polarizing optics such as polarizers and waveplates, was minor.

Supplementary Note 3

Numerical investigation

In this section, we numerically investigate the effect of the uneven transmittance of objective lenses on the degradation of the axial resolution of an RPM. We start from the interferogram presented in Eq. (1) in the main text. To mimic the decrease in the double transmitting amplitude transmittance of an objective lens, we model $t_S(k_\rho) = t_R(k_\rho) = \left(1-\beta k_\rho^2\right)$, where k_ρ is the radial component of wavevector $k = nk_0 = \sqrt{k_\rho^2 + k_z^2}$ with n the refractive index of the immersion medium and $k_0 = \dfrac{\lambda_0}{2\pi}$, and β is a constant representing the degree of decrease in the transmittance. Although the transmittances of the two objective lenses are different in general, we assume that they are identical just for simplicity, which is a reasonable assumption from the similar profiles presented in Figs. 2 (a) and 2 (b) in the main text. Then, from Eq. (1) in the main text, the contribution of the input wave with wavevector $\vec{k}$ to the final interferogram can be expressed as

$$I(\vec{k};\Delta z) = I_S + I_R + 2\eta(k_\rho)\left|E_S^0\right|\left|E_R^0\right|\cos\left(2k_z\Delta z + k_x^R x + \phi_u(\vec{k})\right), \tag{S4}$$

where $\eta(k_\rho) = \left(1-\beta k_\rho^2\right)^2$ is the interference contrast. By assuming the best match of the two arms, the phase term $\phi_u(\vec{k})$ can be neglected. Then Eq. (S4) is further simplified as

$$\begin{aligned} I(k_\rho;\Delta z) &= I_S + I_R + 2\eta(k_\rho)\left|E_S^0\right|\left|E_R^0\right|\cos\left(2k_z\Delta z + k_x^R x\right) \\ &= I_S + I_R + 2\eta(k_x,k_y)\left|E_S^0\right|\left|E_R^0\right|\cos\left(2n\Delta z\sqrt{k_0^2 - k_x^2 - k_y^2} + k_x^R x\right), \end{aligned} \tag{S5}$$

In the second line in Eq. (S5), the relations $k_z = nk_{0z} = n\sqrt{k_0^2 - k_{0\rho}^2}$ and $k_{0\rho}^2 = k_{0x}^2 + k_{0y}^2$ are used. The final interferogram with the scanning of $\left(k_x, k_y\right)$ is obtained by the integration of the expression in Eq. (S5) over the entire NA of the objective lens. For the numerical investigation, we digitize the *k*-space and use a summation instead of the integration as [2]

$$I_{\text{total}}(\Delta z)=\sum_{i}^{N} I_i(k_x^i,k_y^i;\Delta z)=\sum_{k_x^i,k_y^i}^{N}\left(2+2\eta(k_\rho)\cos\left(2n\Delta z\sqrt{{k_0}^2-{k_x^i}^2-{k_y^i}^2}+k_x^R x\right)\right). \qquad \text{(S6)}$$

In Eq. (S6), for further simplicity, we assume that $\left|E_S^0\right|=\left|E_R^0\right|$. With a certain β, we then calculate the sum in Eq. (S6) in numerically generated k-space and attain the interferogram. By repeating the calculation with different Δz, a PSF is obtained. By a Gaussian fitting, a FWHM is determined as the axial resolution of the RPM.

The results are presented in Fig. S3. The decrease of η over the NA is depicted with several values of β in Fig. S3 (a). As β increases, the more decrease at the higher NA side in η. The change of the axial resolution depending on various β is shown in Fig. S3 (b). Because larger β means more drop in the transmittance, the graph confirms that the axial resolution degrades more severely as the transmittance of the objective lens shows more reduction. This result agrees well with the observation in our RPM.

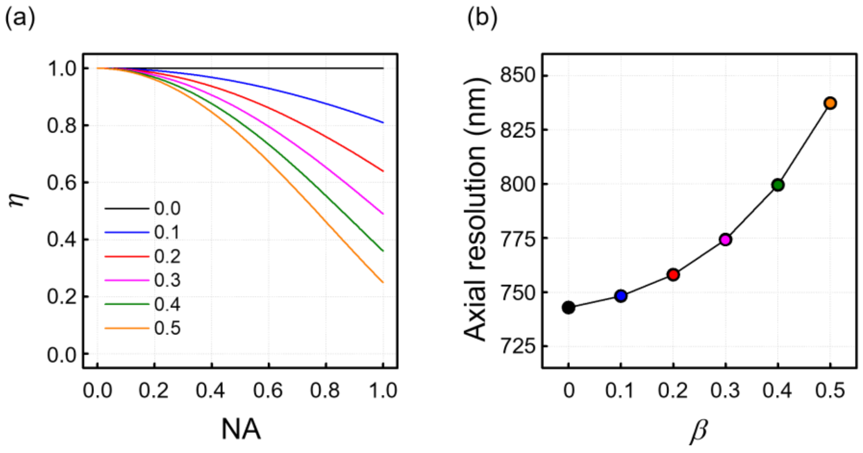

Fig. S3. Numerical investigation on the degradation of the axial resolution. (a) Interference contrast η along the NA of the objective lens. The values of β used for the calculation are listed in the graph with the corresponding colors. (b) Change on the axial resolution calculated using Eq. (S6) with η curves in (a). Each dot has the same color with the curve in (a) used for the calculation.

Supplementary Note 4

Phantom experiment

We made a sample using an agarose gel as a host medium and magnetic beads as scatterers. The size of magnetic beads (Spherotech, Inc.) ranged from 400 to 690 nm. We prepared an agarose gel with a dilution ratio of 1:50 and put the magnetic beads in it. Then we mixed the medium and the scatterers so well that the beads were randomly suspended in the gel. After stabilization, we sought clusters of two beads that were located so close to each other along the *z*-axis that the individual particles were not or barely resolved. We acquired multiple depth images for several clusters at every 20 nm. We repeated the measurement without and with the AM control to compare the axial resolving power between the two cases. One result is shown in Fig. 4 in the main text and the same data is presented in Fig. S4 (a). The insets show the *x*-*z* images of the cluster and the plots are the projected profiles of the *x*-*z* images onto the *z*-axis. The solid lines are fitting curves with two-peak Gaussian functions. In each figure in Fig. 4, the left graph shows the intact case and the right one presents the case with the AM control. By comparison, we found that the AM control made our method more resolve the axial distribution.

Here we show an extra result obtained with another bead cluster and further analysis. We repeated the same measurement for a different bead cluster as shown in Fig. S4 (b). In this time, as seen in the profile, the two beads were so close to each other that those were distinguishable before the AM control (left) due to the significant overlap. But after the AM control, the existence of two particles was identified and the two particles were barely resolved (right).

For more comparison, we applied deconvolution to the depth images using ImageJ™ and redrew the projected profiles. The deconvolution results for Figs. S4 (a) and S4 (b) are presented in Figs. S4 (c) and S4 (d), respectively. After the deconvolution, the beads were more well resolved in the intact and the controlled cases in Fig. S4 (c), and the effect of AM control is shown more dramatically.

In Fig. S4 (d), the deconvolution made the two beads more distinguishable in the controlled case (right) as did for the first cluster in Figs. S4 (a) and S4 (c). However, even after the deconvolution, the beads were still not resolved in the intact case due to the complete overlap between the two particles (left of Fig. S4 (d)). In the case of the first cluster, the two bead profiles were barely separate in Fig. S4 (a), thus the deconvolution could make an effect in Fig. S4 (c). But in the case of the second cluster, the two profiles were entirely overlapped initially (left of Fig. S4 (b)), and consequently, the deconvolution could not make any difference (left of Fig. S4 (d)). In contrast, the AM control made the two beads distinguishable by narrowing the individual bead profiles as shown on the right side in Fig. S4 (b), and then the profiles became more resolvable after applying the deconvolution as presented in the right side of Fig. S4 (d). The deconvolution could not play a role until the fundamental improvement was made in the object profile by the AM control. For both the bead clusters, we confirmed that the AM control reduced the particle profiles and indeed enhanced the axial resolving power in our RPM.

As briefly mentioned in the main text, the measured widths were larger than the ideal case, the ones expected from the axial resolution measured from the mirror experiment. This discrepancy was caused by the mismatch of the medium in the two arms. The beads were suspended in an agarose gel in the sample arm, but there was only water in the reference arm. The small refractive index difference between the agarose and water caused additional angle-dependent phase retardations in the illumination plane wave. This resulted in an aberration between the two arms and disrupted the condition for the SAI process. This phenomenon is similar to that known as a sample-induced aberration which is commonly observed in high-resolution microscopy [3-5]. Consequently, the particle distributions were broadened in the axial direction.

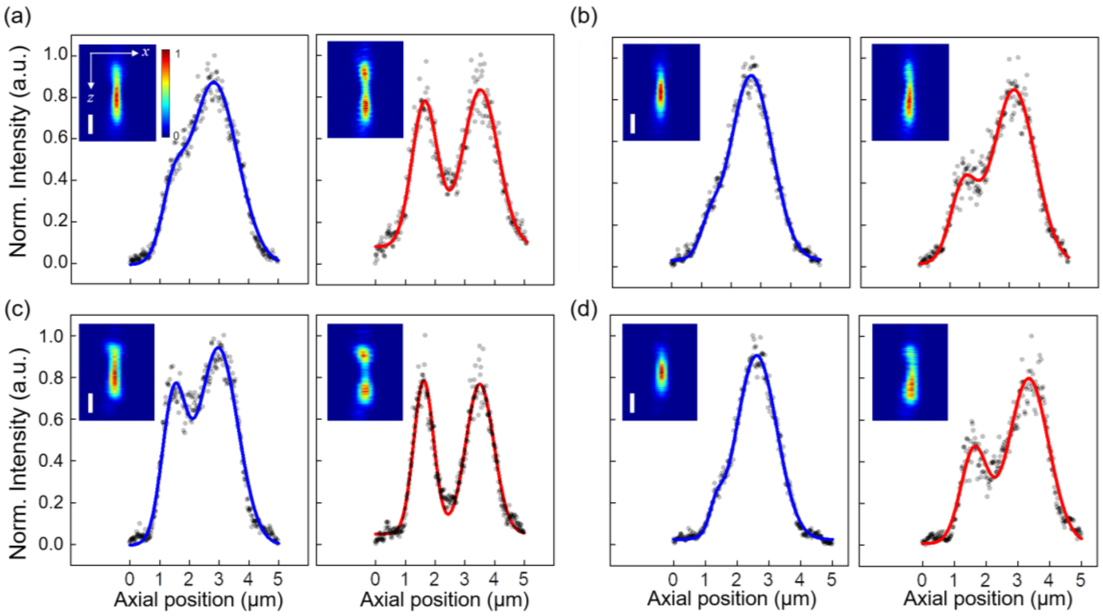

Fig. S4. Enhancement of axial resolution in a phantom sample. (a) Projected profiles of the 2-D images of a bead cluster shown in the insets without (left) and with (right) the AM control. Insets: the *x-z* images of the bead cluster. This data is the same as that presented in Figs. 4(f)-4(i) in the main text. (b) The same as (a), but for a different bead cluster. (c) Deconvoluted profiles for the data in (a). (d) Deconvoluted profiles for the data in (b). Scale bars: 1 μm, colorbar: normalized intensity. The solid lines are fitting curves with two-peak Gaussian functions.

Supplementary References

1. D. A. Tichenor, and J. W. Goodman, "Coherent Transfer Function*," J. Opt. Soc. Am. **62**, 293-295 (1972).
2. M. G. Hyeon, T. D. Yang, J.-S. Park, K. Park, Y. G. Kang, B.-M. Kim, and Y. Choi, "Reflection phase microscopy by successive accumulation of interferograms," ACS Photonics **6**, 757-766 (2019).
3. M. Schwertner, M. J. Booth, and T. Wilson, "Characterizing specimen induced aberrations for high NA adaptive optical microscopy," Opt. Express **12**, 6540-6552 (2004).
4. D. Burke, B. Patton, F. Huang, J. Bewersdorf, and M. J. Booth, "Adaptive optics correction of specimen-induced aberrations in single-molecule switching microscopy," Optica **2**, 177-185 (2015).
5. M. Kim, Y. Jo, J. H. Hong, S. Kim, S. Yoon, K.-D. Song, S. Kang, B. Lee, G. H. Kim, H.-C. Park, and W. Choi, "Label-free neuroimaging in vivo using synchronous angular scanning microscopy with single-scattering accumulation algorithm," Nat. Commun. **10**, 3152 (2019).
